# Flavonoid-enriched extract from *Hippophae rhamnoides* seed reduces high fat diet induced obesity, hypertriglyceridemia, and hepatic triglyceride accumulation in C57BL/6 mice

**DOI:** 10.1080/13880209.2016.1278454

**Published:** 2017-03-01

**Authors:** Xin Yang, Qian Wang, Zeng-run Pang, Meng-ran Pan, Wen Zhang

**Affiliations:** a School of Life Sciences, East China Normal University, Shanghai, P.R. China;; b Institute of Oncology, Shanghai 9th People's Hospital, Shanghai Jiao Tong University School of Medicine, Shanghai, P.R. China

**Keywords:** Obese mice, PPARγ, PPARα, inflammation

## Abstract

**Context:** Flavonoid-enriched extract from *Hippophae rhamnoides* L. (Elaeagnaceae) seed (FSH) has shown beneficial effects in anti-hypertension and lowering cholesterol level. However, evidence for its efficacy in treating obesity is limited.

**Objective:** We sought to determine if FSH can reduce body weight and regulate lipid metabolism disorder in high fat diet (HFD)-induced obese mouse model, and to investigate potential molecular targets involved.

**Materials and methods:** C57BL/6 mice were fed with HFD for 8 weeks to induce obesity. The modeled mice were divided into four groups and treated with vehicle, rosiglitazone (2 mg/kg), low (100 mg/kg) and high (300 mg/kg) dose of FSH, respectively. Normal control was also used. The treatments were administered orally for 9 weeks. We measured the effect of FSH on regulating body weight, various liver and serum parameters, and molecular targets that are key to lipid metabolism.

**Results:** FSH administration at 100 and 300 mg/kg significantly reduced body weight gain by 33.06 and 43.51%, respectively. Additionally, triglyceride concentration in serum and liver were decreased by 15.67 and 49.56%, individually, after FSH (300 mg/kg) treatment. Upon FSH (100 and 300 mg/kg) treatment, PPARα mRNA expression was upregulated in liver (1.24- and 1.42-fold) and in adipose tissue (1.66- and 1.72-fold). Furthermore, FSH downregulated PPARγ protein level both in liver and adipose tissue. Moreover, FSH inhibited macrophage infiltration into adipose tissues, and downregulated TNFα mRNA expression in adipose tissue (38.01–47.70%).

**Conclusion:** This effect was mediated via regulation of PPARγ and PPARα gene expression, and suppression of adipose tissue inflammation.

## Introduction

Obesity develops when energy intake exceeds energy expenditure. Studies have shown that fat distribution also contributes to metabolic risk (Spiegelman & Flier 2001; Gesta et al. 2007). White adipose tissue (WAT) is positively related to obesity. Increased fat mass, particularly increase in adipocyte number and size, is also a characteristic of obesity. In adipocytes, approximately 90% of cell volume is lipid droplet; the nucleus and the thin cytoplasmic rim are pushed to the periphery of the adipocytes (Fruhbeck et al. 2001). WAT expands during childhood obesity due to adipocyte hypertrophy and hyperplasia. By contrast, adults likely have a fixed number of adipocytes, and changes in fat mass are mostly secondary to changes in fat-cell volume (Christodoulides et al. 2009). Excess energy results in adipocyte hypertrophy. To accommodate lipids, adipocytes can change diameter by 20-fold and volumes by several thousand fold. Hypertrophied adipocytes secrete increased amounts of chemoattractants such as monocyte chemotactic protein 1 and interleukin (IL)-8, which attracts monocytes into the adipose tissue where they differentiate into macrophages (van de Woestijne et al. 2011). Obesity is associated with a chronic low-grade inflammatory state, as characterized by increased numbers of macrophages, of adipose tissue. These infiltrating macrophages release pro-inflammatory cytokines, such as tumor necrosis factor (TNF)-α, IL-6, and IL-1β, all of which promote lipolysis and insulin resistance in the adipocytes (Weisberg et al. 2003; van de Woestijne et al. 2011). Therefore, suppressing adipocyte hypertrophy and decreasing the numbers of infiltrating macrophages are important for alleviating inflammation and lipid metabolic disorder in obesity.

Peroxisome proliferator-activated receptor γ (PPARγ) is the major regulator of lipid storage in WAT (Anghel & Wahli 2007). As another important member of the PPAR family, PPARα regulates the expression of a number of genes critical for lipid and lipoprotein metabolism, including mitochondrial and peroxisomal fatty acid oxidation, as well as fatty acid uptake and transport (Fruchart 2009). Accordingly, modulation of PPARγ and PPARα is very important for tackling lipid metabolic disorder associated with obesity.

*Hippophae rhamnoides* L. (Elaeagnaceae) bush, which produces delicious and nutritious orange-colored berries, is locally called ‘Shaji’ in China. The berries of *H. rhamnoides* are nutritious and have been widely used by local residents as food and folk medicine for a long time. *H. rhamnoide*s has been reported to possess a wide spectrum of health beneficial activities, such as antioxidant, antiulcer, immunomodulatory hepatoprotective, and cardioprotective properties (Suleyman et al. 2001; Geetha et al. 2002; Cheng et al. 2003; Basu et al. 2007; Maheshwari et al. 2011). Our previous studies reported that flavonoid-enriched extract from the seed of *H. rhamnoide*s (FSH) has antihypertensive effect on sucrose-fed rats (Pang et al. 2008). FSH also effectively decreases serum total cholesterol (TC) and glucose concentrations in hypercholesterolemic mice, which is induced by feeding high fat diet containing cholesterol and bile salt (HFCBD) (Wang et al. 2011). Although this mouse model exhibits elevated circulating cholesterol, HFCBD feeding does not affect the body weight of treated mice and causes remarkable decline in circulating triglyceride (TG). Accordingly, the effect of FSH on obesity and triglyceride metabolism remains poorly understood. The obese C57BL/6 mouse model induced by high fat diet (HFD) has been used widely as an animal model for human obesity, and HFD induced obesity in this strain manifests the typical features of human metabolic syndrome (Okabe et al. 2011). Therefore, this study was performed to investigate the effect of FSH on obesity using HFD induced obese C57BL/6 mice. To elucidate the mechanism underlying the regulation of lipid metabolism by FSH in obese mice, we examined its effects on the expression of genes critical for lipid and lipoprotein metabolism, including lipid storage, fatty acid transport and oxidation. Furthermore, the expression of inflammatory cytokines in these mice was also examined.

## Materials and methods

### Plant material

The seeds of *H. rhamnoides* were collected in Jining, Inner Mongolia in February 2013, and authenticated by Professor Hong-qing Li, School of Life Science, East China Normal University, Shanghai. A voucher specimen (No.: Sha J. S. Inner M. 2013) was deposited in the herbarium of East China Normal University (Shanghai, China).

### Preparation of FSH and HPLC analysis

Extraction was performed in accordance with previously described methods (Pang et al. 2008). In brief, crushed powder of seeds was boiled three times (6 h each) in petroleum ether at 70 °C. The powder was air dried in a ventilated cabinet and then incubated with 700 mL/L ethanol at 80 °C four times (2 h each) to extract crude total flavones. The crude total flavonoids were then applied to a D101 macroporous resin column and eluted with ethanol from 300 mL/L to 500 mL/L at the speed of 2 mL/min. The fractions containing flavonoids were collected and reclaimed using vacuum rotary evaporation. The achieved concentrated liquids were further freeze dried to produce a solid FSH powder. The average percentage yield of FSH was found to be 3.08% w/w, and the total flavonoid content in the resulting FSH powder was (794.3 ± 10.2) g/kg determined using a colorimetric aluminum nitrate method as described in the Chinese Pharmacopoeia (Chinese Pharmacopoeia Commission 2010).

**Figure 1.**
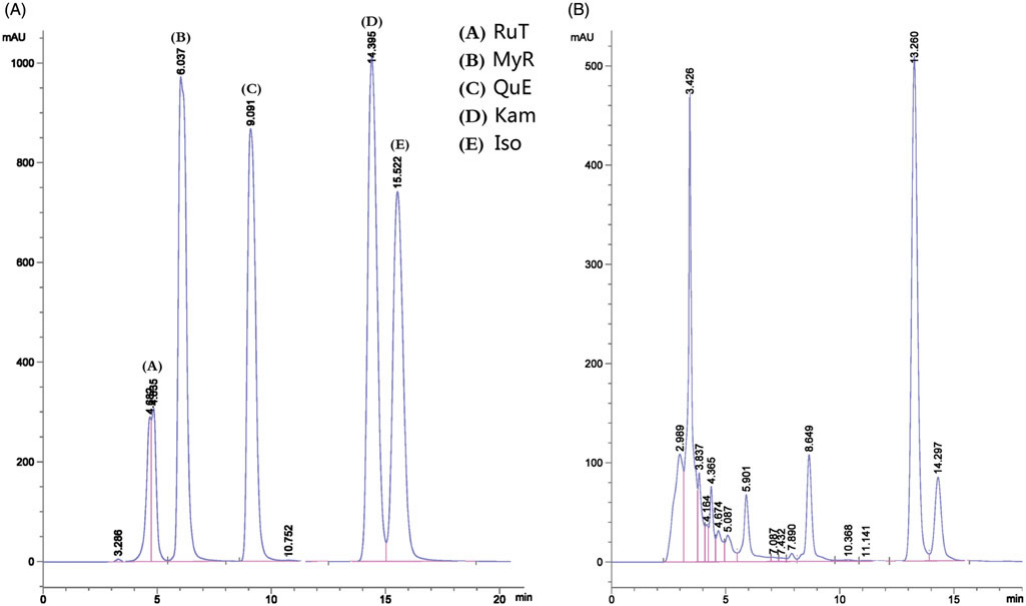
Chromatograms of the standard sample (A) and the hydrolyzed sample (B) analyzed through HPLC. RuT: Rutin; MyR: Myricetin; QuE: Quercetin; Kam: kaempferol; Iso: Isorhamnetin.

In order to analyze the main flavonoid aglycones, the extract was hydrolyzed by 1.5 M hydrochloric acid at 90 °C for 2 h and further analyzed using an HPLC system (HP1100, Agilent). Chromatography was performed on a C18 column (250 mm ×4.6 mm, No.LAAI-KR006, Kromasil). Mobile phase was methyl alcohol: 0.4% phosphoric acid solution at a ratio of 60: 40. The flow rate was 1 mL/min at room temperature, and detection was performed at 368 nm. The results are shown in Figure 1. The myricetin, quercetin, kaempferol, and isorhamnetin contents of the total flavonoids were 3.47 ± 0.74, 5.43 ± 0.63, 34.82 ± 0.16, and 6.03% ± 0.19%, respectively.

### Animals and induction of obesity model

C57BL/6 mice (male, eight weeks old, specific pathogen-free) were obtained from the Central Lab Animal, East China Normal University [license key is SYXK (Shanghai) 2010-0094]. Animal procedures were approved by the Experimental Animal Ethical Review Committee, East China Normal University. All of the mice were housed in plastic cages (five mice/cage) with free access to drinking water and a strict diet under controlled conditions of humidity (50% ± 10%), light (12 h light/dark cycle), and temperature (23 ± 2 °C).

Ten C57BL/6 mice were set as the normal control (NC) group and fed with a normal diet (ND). The other mice were fed HFD (45% fat calories; Shanghai SLAC Laboratory Animal Co., Ltd.). The nutritional composition of these diets is shown in Table 1. After eight weeks, the mice fed with HFD exhibited 20% higher weight gain than those fed with normal diet. The HFD-induced obese mice were subdivided into four groups (10 mice/group) according to body weight (BW). One group served as the HFD control group (HFD group), and the other groups received drug or extract treatments. The mice in the two control groups continued to receive their original diet of ND or HFD, with the addition of equal volumes of 0.5% carboxymethyl cellulose (CMC, Aladdin, Shanghai, China) solution. The treatment groups were fed HFD with either rosiglitazone at a dose of 2 mg/kg body weight (BW) or with FSH at two different doses (100 and 300 mg/kg BW) by intragastric administration once a day in the morning for a subsequent nine weeks. Rosiglitazone and FSH were resuspended in 0.5% CMC solution. Mouse body weight was measured at an interval of 3 d.

**Table 1.** Composition of the diets fed to the mice.

Ingredient (g)	Normal diet (M01-F52)	High-fat diet
Moisture (%) ≤	10	−
Crude protein (%) ≥	20.5	−
Crude fat (%) ≥	4	−
Crude fiber ≤	5	−
Crude ash ≤	8	−
Calcium salt (%)	1.0–1.8	−
Total phosphorus	0.6–1.2	−
Lysine (%) ≥	1.32	−
Methionine/Cystine (%) ≥	0.78	−
Salt (%)	0.4	−
N free extract (NFE)	48.4–49.5	−
Normal diet (%)	−	49.5
Lard (%)	−	20.4
Sucrose (%)	−	15
Casein (%)	−	12.3
Gunk (%)	−	2
Maltodextrin (%)	−	0.8
	345.0 Kcal/100 g	460.5 Kcal/100 g

### Oral glucose tolerance test (OGTT)

OGTT was performed after six weeks of FSH administration. All of the mice were allowed to fast overnight for 14 h. Oral treatment of rosiglitazone (2 mg/kg BW), FSH (100 and 300 mg/kg BW), or 0.5% CMC was administered 30 min before glucose was orally administered (2.0 g/kg BW). Blood samples were obtained from the tail vein to monitor blood glucose (Glu) content 30 min before glucose was orally administered, and 0, 30, 60, 90, and 120 min after glucose was administered using a glucometer (Roche Diagnostics GmbH, Mannheim, Germany). The area under the curve (AUC) was calculated using the following equation: AUC = (basal glycemia + glycemia 0.5 h) × 0.25 + (glycemia 0.5 h + glycemia 1 h) × 0.25 + (glycemia 1 h + glycemia 2 h) × 0.5.

### Biochemical analysis and organ weight

After nine weeks of FSH treatment, fasting glucose (Glu) concentrations were measured using a glucometer. Then the animals were sacrificed through cervical dislocation after 12 h of overnight fasting. Blood samples were collected, and liver, spleen, kidney, and heart were weighed. Serum was separated through centrifugation, and serum samples were immediately frozen at −80 °C until use in subsequent assays. Serum TG, TC, and high-density lipoprotein cholesterol (HDL-C) concentrations were measured using commercial kits according to the manufacturer’s instructions (Nanjing Jiancheng Bioengineering Institute, Nanjing, China).

### Histological and immunohistochemical (IHC) analysis of liver and epididymal WAT

Liver and WAT samples were immediately retrieved and stored in liquid nitrogen until the samples were assayed. TG, TC, and protein concentrations in liver homogenates were measured using commercial kits (Nanjing Jiancheng Bioengineering Institute, Nanjing, China).

Small pieces of liver and epididymal WAT were dissected, washed in saline, fixed in 10% formalin, and embedded in paraffin. Tissue sections were cut at a thickness of 5 μm and stained with hematoxylin and eosin. F4/80 antibody was used to stain macrophages in the adipose tissue. The approximate area of epididymis fat cells was estimated using Image-Pro plus 6.0 (Silver Spring, MD). 

### Quantitative reverse transcription polymerase chain reaction (qRT-PCR) for gene expression

Total RNA was extracted from the epididymal WAT and liver (*n* = 6) and reverse-transcribed to cDNA using a reverse transcription kit (RR037A, TaKaRa, Otsu, Shiga 520-2193, Japan). Approximately 1000 ng of total RNA was used in each subsequent qRT-PCR step. The primer pairs used in the analysis are shown in Table 2. qRT-PCR was performed under the following conditions: denaturation at 95 °C for 10 min, and 40 cycles of annealing at 95 °C for 15 s, and extension at 60 °C for 60 s.

**Table 2.** qRT-PCR primer sequences.

Gene name	Forward primer	Reverse primer
β actin	CGCTCGTTGCCAATAGTG	GCTGTGCTATGTTGCTCTAG
LDLR	AGTGGCCCCGAATCATTGAC	CTAACTAAACACCAGACAGAGGC
PPARα	AGAGCCCCATCTGTCCTCTC	ACTGGTAGTCTGCAAAACCAAA
PPARγ	CAGAATACCAAAGTGCGATCAA	GAGCTGGGTCTTTTCAGAATAATAAG
C/EBPα	AGCAACGAGTACCGGGTACG	TGTTTGGCTTTATCTCGGCTC
FAT	ATGGGCTGTGATCGGAACTG	GTCTTCCCAATAAGCATGTCTCC
GluT 1	CGTGCTCTTCTTCATCTTCACCTAC	GTCTTCAGCAGTTAAGTTCTCAGCC
TNFα	AGAGCTACAAGAGGATCACCAGCAG	TCAGATTTACGGGTCAACTTCACAT

### Protein extraction and Western blotting

Liver and epididymal fat pad samples were homogenized in modified RIPA buffer (50 mmol/L Tris Base 8.0, 1% Triton X-100, 0.5% sodium deoxycholate, 0.1% sodium dodecyl sulfate (SDS), phosphatase inhibitor cocktail (Biotool, Houston, TX, USA, Cat. No.: B15001), and protease inhibitor cocktail (Biotool, Houston, TX, USA, Cat. No.: B14001)). The supernatant was then centrifuged at 2000 *g* for 25 min at 4 °C, and the resulting supernatant was collected. Aliquots (50,000 ng protein) of the protein were separated through SDS-polyacrylamide gel electrophoresis and transferred to nitrocellulose filter membranes (ExCell Bio, Qingpu District, Shanghai, China, Cat. No.: CS011-0001). Membranes were blocked with 5% (w/v) bull serum albumin (BSA; MP Biomedicals New Zealand Limited, Henderson, Auckland, New Zealand, Cat. No.: 0218054980) in Tris-buffered saline containing 0.1% Tween 20 (TBS-T) and incubated with the following antibodies in TBS-T containing 5% BSA: anti-rabbit PPARγ (ProteinTech Group, Inc., Chicago, IL, USA; 1:1000). After incubation was performed overnight, the membranes were washed with TBS-T and then incubated with anti-rabbit IgG secondary antibody (Gibco BRL, Gaithersburg, MD) in 5% BSA in TBS-T. After the membranes were washed, the immune complexes were detected using a chemiluminescence system (LI-COR, Lincoln, NE).

### Statistical analysis

Data are expressed as mean ± standard error of the mean (S.E.M) and were analyzed using SPSS 13.0 software. Significant differences among multiple groups were analyzed by one-way analysis of variance (ANOVA) followed by Duncan’s or Dunnett’s T3 multiple range test. *p* < 0.05 and *p* < 0.01 were considered statistically significant and extremely significant between groups, respectively.

## Results

### Body and organ weights

**Figure 2.**
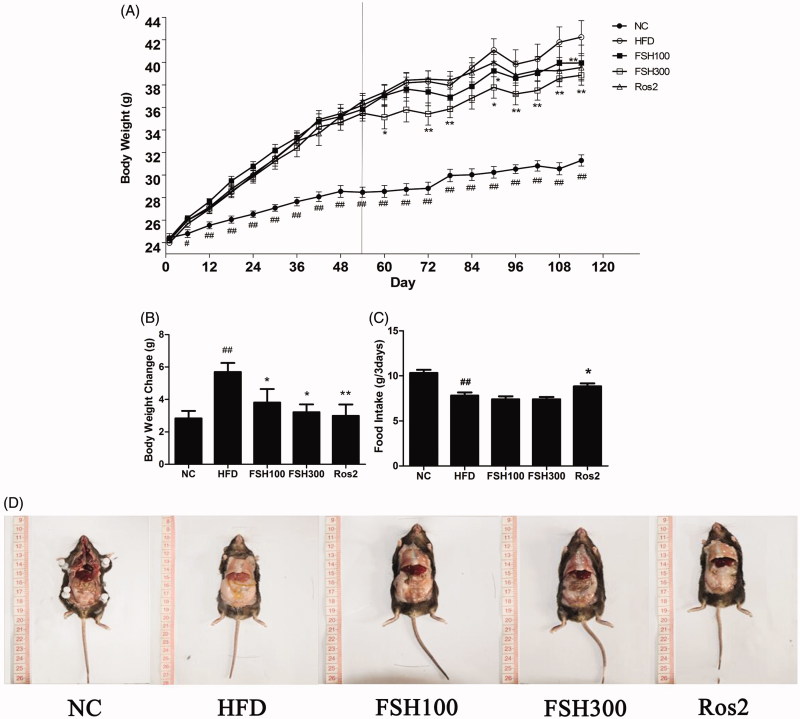
Effect of FSH on body weight and food intake in HFD-induced obese mice. (A) Body weight during the experiment. (B) Change of body weight between the 9th week and 17th week. (C) Average food intake per mouse was recorded at an interval of 3 d. (D) Abdominal adipose tissue. Values are expressed as mean ± S.E.M (*n* = 10). *p < 0.05, **p < 0.01, vs. the HFD group, respectively; ##*p* < 0.01, vs. the NC group.

Body weight was recorded during the experimental period. At the beginning of rosiglitazone and FSH treatment, body weight of HFD-induced obese mice evidently increased compared with that of normal mice (*p* < 0.01, Figure 2(A)). At the end of the experiment, body weight gains of the mice fed with HFD plus FSH at doses of 100 and 300 mg/kg were 33.06 and 43.51% lower, respectively, compared to those of mice fed with HFD alone (*p* < 0.05, Figure 2(B)).

In addition, the relative weights of liver, kidney, heart, and spleen were measured (Table 3). The liver weights of Ros-treated mice significantly increased (*p* < 0.01) compared to the controls, which indicated that Ros might lead to liver hyperplasia.

**Table 3.** Effect of FSH on organ weights.

Organ weight g/100 g BW	NC	HFD	FSH100	FSH300	Ros2
Liver	3.60 ± 0.26	3.35 ± 0.37	3.38 ± 0.66	3.31 ± 0.30	5.94 ± 0.74**
Kidney	1.11 ± 0.06	0.96 ± 0.09	1.06 ± 0.15	0.97 ± 0.06	0.99 ± 0.12
Heart	0.52 ± 0.06	0.35 ± 0.05##	0.38 ± 0.04	0.41 ± 0.08	0.38 ± 0.04
Spleen	0.20 ± 0.05	0.16 ± 0.02#	0.17 ± 0.03	0.18 ± 0.02	0.17 ± 0.03

Values are expressed as mean ± S.E.M. (*n* = 10).

** *p* < 0.01 vs. HFD group

# *p* < 0.05

## *p* < 0.01 vs. NC group.

### Lipid profiles in serum and liver, fasting blood glucose and OGTT

**Figure 3.**
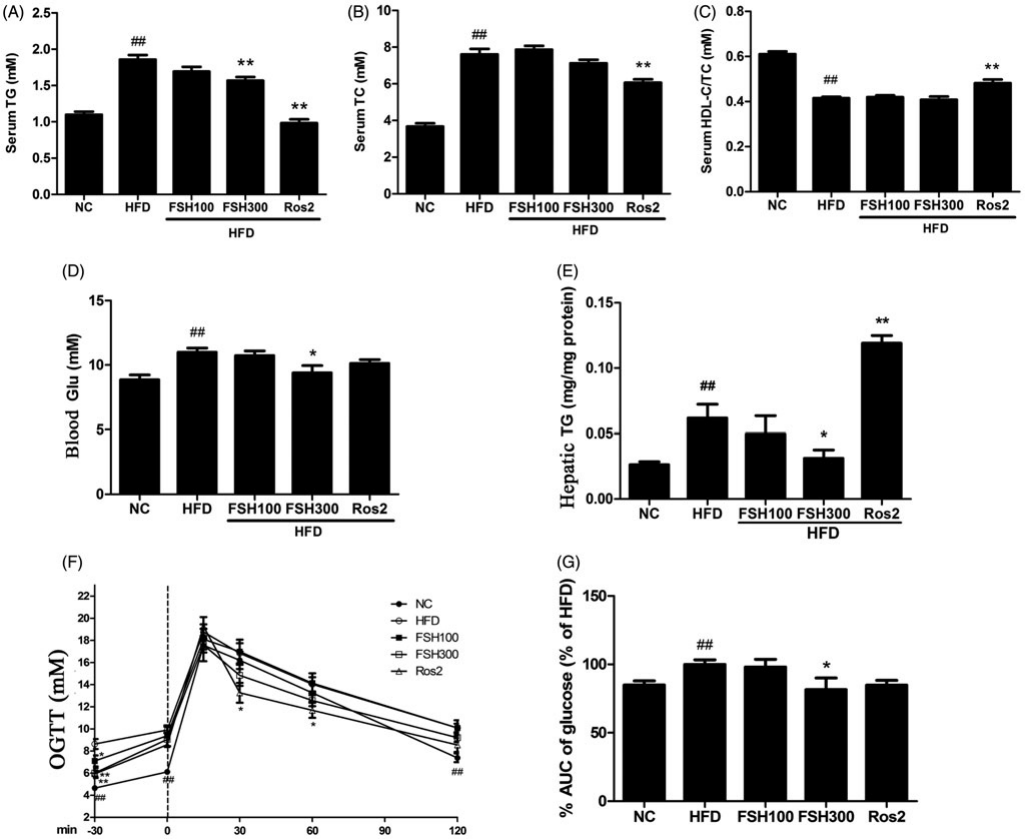
Effect of FSH on serum lipid profiles, blood glucose, hepatic TG, and OGTT. (A) Serum TG. (B) Serum TC. (C) Serum HDL-C/TC. (D) Fasting blood Glu. (E) Hepatic TG. (F) Blood glucose concentrations during an OGTT. (G) Area under the curve of blood glucose in OGTT. Values are expressed as mean ± S.E.M. (*n* = 10). *p < 0.05, **p < 0.01, vs. HFD group, respectively; ##*p* < 0.01, vs. NC group.

Serum lipid profiles and hepatic TG were determined to investigate the possible role of FSH in lipid metabolism. Figure 3(A,B) show that serum TG and TC concentrations markedly increased in HFD-fed mice (*p* < 0.01 vs. normal mice), and this abnormal TG was decreased by 15.67% after nine weeks of 300 mg/kg FSH administration (*p* < 0.01 vs. HFD control). However, FSH feeding did not significantly affect serum TC (Figure 3(B)) or HDL-C concentrations (Figure 3(C)) in obese mice (*p* > 0.05). Hepatic TG concentration was determined when the experiment was completed (Figure 3(E)). The result showed that hepatic TG level remarkably increased by 2.35-fold in the HFD control group compared with that of the NC group (*p* < 0.01). However, hepatic TG of the 300 mg/kg FSH treated mice was decreased by 49.56% compared with that of the HFD-fed control mice (*p* < 0.05).

Figure 3(D) showed that fasting blood glucose concentration increased after induction of HFD (*p* < 0.01 vs. normal mice). Blood glucose concentrations were lower in the FSH treatment group (300 mg/kg) by 14.55% (*p* < 0.05) compared to the HFD control.

To investigate the effect of FSH on glucose tolerance, we performed OGTT after six weeks of FSH treatment. As shown in Figure 3(F), blood glucose concentrations were significantly higher in the HFD control group than in the NC group at all of the time points after glucose was loaded (*p* < 0.01). This result suggested an impaired glucose tolerance (IGT) state. Such abnormal increase in blood glucose concentration was significantly prevented by FSH and Ros treatment. At 15, 60, and 120 min after oral glucose was administered, blood glucose of the FSH300 group was reduced by 16.32%, 18.13%, and 18.78%, respectively, compared to the HFD control group (*p* < 0.01). Additionally, FSH300 group showed significant reduction in AUC (*p* < 0.05). These results indicated that FSH alleviated glucose intolerance in HFD-induced obese mice.

### Histological analysis of WAT and liver tissues

**Figure 4.**
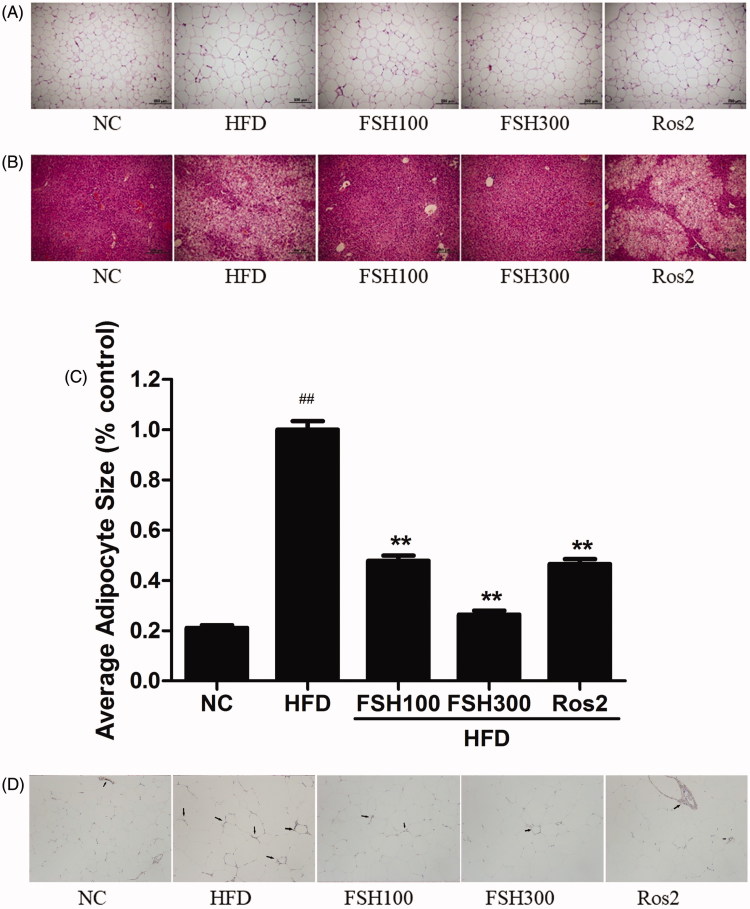
Histological analysis of epididymial WAT and liver tissue. (A) Photomicrographs of epididymal WAT stained with HE (100×). (B) Photomicrographs of liver tissues stained with HE (100×). (C) Adipocyte size (% control, *n* = 500; 100×). (D) Photomicrographs of epididymal white adipose tissues with F4/80 antibody. Values are expressed as mean ± S.E.M (*n* = 10). *p < 0.05, **p < 0.01, vs. HFD group, respectively; ##*p* < 0.01, vs. NC group.

The results of HE staining of epididymal fat pad and liver tissue in each group are shown in Figure 4(A,B). Average adipocyte size (% control) was estimated using Image-Pro Plus 6.0, and the results were showed in Figure 4(C). Adipocyte size of the epididymal fat pad in the HFD group was significantly higher than that of the epididymal fat pad in the NC group (*p* < 0.01). However, the sizes of adipocytes were considerably lower in the FSH100 and FSH300 groups compared with those in the HFD group (*p* < 0.01). These findings indicated that the anti-obesity activity of FSH may be attributed partly to decrease in volume of fat cells. As shown in representative images of HE-stained cells in Figure 4(B), FSH suppressed lipid accumulation in the liver. By contrast, Ros enhanced lipid accumulation in the liver. Immunohistochemical images of WAT stained by F4/80 indicated that FSH can reduce the infiltration of macrophages into the adipose tissue of obese mice (Figure 4(D)).

### mRNA expression of lipid metabolism-related genes in liver and WAT

To better understand the molecular mechanism by which FSH mediates its beneficial effects on obese mice, the expression of genes involved in lipid metabolism was investigated.

**Figure 5.**
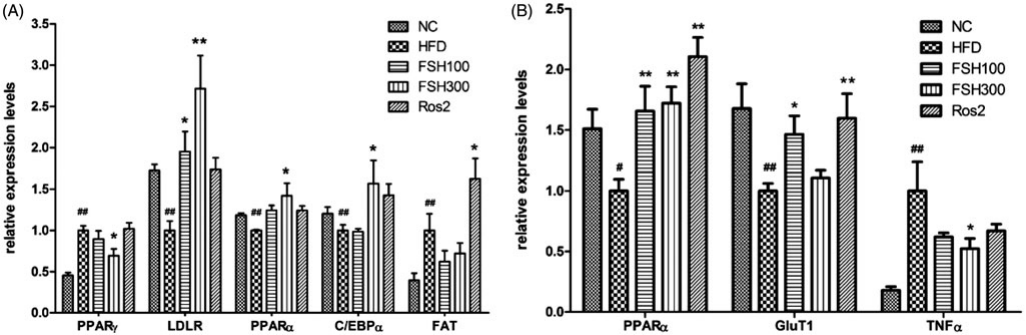
Effects of FSH on mRNA expression in the liver (A) and epididymal WAT (B) of HFD-induced obese mice. Values are expressed as mean ± S.E.M (*n* = 6). *p < 0.05, **p < 0.01, vs. HFD group, respectively; ##*p* < 0.01, vs. NC group.

As shown in Figure 5(A), 300 mg/kg FSH significantly downregulated mRNA expression of PPARγ in the liver (*p* < 0.05). mRNA levels of PPARα and low-density lipoprotein receptor (LDLR) were upregulated after FSH treatment (*p* < 0.05 or *p* < 0.01). Moreover, CCAAT/enhancer-binding protein α (C/EBPα), an adipogenic marker gene, was also upregulated in the liver of mice treated with 300 mg/kg FSH (*p* < 0.05). The mRNA level of fatty acid translocase (FAT), which is closely related to free fatty acid transport, remained unchanged in the liver after FSH treatment (*p* > 0.05). Consistent with the increased expression in the liver, the mRNA level of PPARα in epididymal WAT was also significantly upregulated by FSH treatment (Figure 5(B), *p* < 0.01).

Additionally, in the WAT of mice treated with FSH at a dose of 100 mg/kg BW, we observed upregulation of glucose transporter 1 (GluT1), which is associated with glucose transport (Figure 5(B); *p* < 0.05). Interestingly, expression of this gene was slightly affected by a high dose of FSH (300 mg/kg BW; *p* > 0.05; Figure 5(B)). It is known that TNFα is closely associated with obesity-induced chronic inflammation. As shown in Figure 5(B), FSH (300 mg/kg BW) significantly suppressed mRNA expression of TNFα in the WAT (*p* < 0.05).

### PPARγ protein expression in the liver and WAT

**Figure 6.**
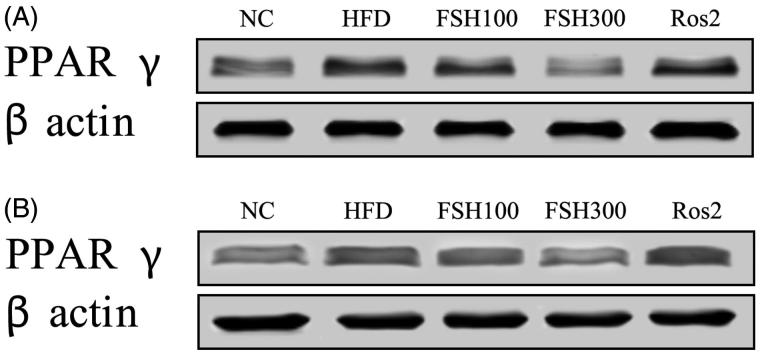
Effects of FSH on the protein expressions of PPARγ in liver (A) and epididymal WAT (B) of HFD-induced obese mice.

Western blotting was performed in this study to investigate the effect of FSH on PPARγ expression. Figure 6 showed that 100 and 300 mg/kg FSH significantly suppressed the protein levels of PPAR γ in the liver (Figure 6(A)) and epididymal WAT (Figure 6(B)). As a PPARγ agonist, Ros significantly upregulated PPARγ expression (Figure 6(A,B)). This result suggested that FSH might elicit effects contrary to those of Ros.

## Discussion

Taken together, our results indicated that FSH exhibited significant anti-obesity, triglyceride-lowering and hypoglycemic effects in HFD-induced obese mice. This extract markedly reduced body weight, TG concentration in serum and liver, and blood glucose level. Our preliminary experiments found that PPARγ expression in adipocytes was downregulated by FSH (data not shown), suggesting that PPARγ might be a target of FSH. Therefore, Ros, a TZD drug that targets PPARγ, was selected as a positive control in this study (Mayerson et al. 2002). Previous studies have reported that Ros causes weight and fatty liver (Hemmeryckx et al. 2013; Sundaresan et al. 2014). However, we found that the BW of mice treated with Ros was lower compared to the controls. Measurement of liver TG level and the results of HE staining showed excess lipid accumulation in liver of Ros-treated mice, which exhibited severe steatosis. Fatty liver is associated with various metabolic disorders (Day & Saksena 2002); as such the weight reduction observed in Ros-treated mice may be partially attributed to the pathological complications of hepatic steatosis.

FSH elicited anti-obesity activity, but FSH administration did not affect the amount of food intake of the treated mice. In the present study, HFD feeding resulted in adipocyte hypertrophy, whereas FSH decreased the size of adipocytes under HFD-fed condition. This finding further confirmed the anti-obesity effect of FSH. In addition, FSH suppressed the mRNA and protein expression levels of PPARγ. PPARγ is a pivotal coordinator of adipocyte differentiation and lipid storage. It promotes release of FFAs from circulating lipoproteins by regulating lipoprotein lipase expression, and enhances expression of proteins involved in uptake, binding and packaging of FFA/TG. Furthermore, PPARγ also promotes the esterification of FFA into TG and its storage (Anghel & Wahli 2007). In the present study, PPARγ expression was suppressed by FSH treatment. This inhibition led to suppression of lipid storage in WAT, which may be the cause of reduction in size and TG accumulation of adipocytes. Previous studies reported that mice treated with PPARγ partial antagonists such as SR202, GW9662, or BADGE showed decreased TG content in WAT, liver and skeletal muscle, decreased adipocyte size, and increased resistance to HFD-induced obesity (Anghel & Wahli 2007). Therefore, the anti-obesity effect of FSH may be attributed partially to suppression of PPARγ expression. In this study, mRNA expression of hepatic PPARα in mice was significantly upregulated by FSH. It is well known that activated PPARα decreases TG by increasing free fatty acid β-oxidation (Yoon 2009), hepatic lipoprotein lipase expression, and apolipoprotein V (apoV) expression (Jeong & Yoon 2009). In addition, it decreases TG by decreasing apolipoprotein CIII (apoCIII) expression (Fruchart 2009). Thus, our findings suggest that FSH may also regulate disorders of lipid metabolism through PPARα, thereby decreasing serum TG concentration in the obese mouse model.

It is known that accumulation of TG in the liver by HFD causes nonalcoholic fatty liver disease (Koeck et al. 2014). In this study, FSH markedly reduced hepatic TG concentrations and our observations are consistent with those of HE-stained liver biopsy. This result indicates that FSH may prevent excess lipid accumulation in tissues and thereby ameliorate fatty liver. The results of OGTT and blood glucose analysis indicated that long-term HFD treatment induced increase in serum glucose concentration and impaired glucose tolerance. These abnormalities could be reversed by administering FSH. Thus, our findings revealed that FSH exhibits hypoglycemic activity, which may prevent the development of diabetes. Furthermore, qRT-PCR results revealed that Glut1 expression was upregulated by FSH, suggesting that glucose intake may contribute to blood glucose reduction.

In obesity, the enlarged adipose tissue is infiltrated by a large number of macrophages, due to which production of pro-inflammatory adipokines such as MCP-1, IL-6, and TNFα increases. Overproduction of pro-inflammatory adipokines contributes to chronic inflammation and plays an important role in the development of insulin resistance (MacDougald & Mandrup 2002; Yu et al. 2013). In this investigation, macrophage infiltration in WAT of obese mice increased significantly, but FSH administration significantly decreased the number of macrophages that infiltrated WAT. Moreover, FSH treatment decreased the mRNA levels of a major mediator of obesity-related inflammation, TNF-a, which promotes inflammatory processes and induces insulin resistance. Thus, these results indicate that FSH treatment inhibits chronic inflammation of WAT, and suggest that the anti-inflammatory effect of FSH may be useful in ameliorating insulin resistance. Furthermore, our data suggest that the hypoglycemic effect of FSH is caused at least in part by inhibition of chronic inflammation via suppression of macrophage infiltration in WAT.

## Conclusion

The present study demonstrated that FSH exhibited anti-obesity, triglyceride-lowering and hypoglycemic activities in obese mice. Additionally, FSH markedly inhibited macrophage infiltration into adipose tissues. Further mechanistic study showed that amelioration of lipid metabolism disorder by FSH may be mediated at least in part by suppressing PPARγ expression and upregulating PPARα expression, and inhibiting inflammation in adipose tissues. Thus, FSH exhibits potential for use in food supplements given its therapeutic properties.
